# Association Between Epidural Analgesia and Cancer Recurrence or Survival After Surgery for Renal Cell Carcinoma: A Propensity Weighted Analysis

**DOI:** 10.3389/fmed.2021.782336

**Published:** 2022-01-14

**Authors:** Fang-Yu Yen, Wen-Kuei Chang, Shih-Pin Lin, Tzu-Ping Lin, Kuang-Yi Chang

**Affiliations:** ^1^Department of Anesthesiology, Taipei Veterans General Hospital, Taipei, Taiwan; ^2^School of Medicine, National Yang Ming Chiao Tung University, Taipei, Taiwan; ^3^Department of Urology, Taipei Veterans General Hospital, Taipei, Taiwan

**Keywords:** epidural analgesia, inverse probability of treatment weighting, recurrence, renal cell carcinoma, survival

## Abstract

Whether epidural anesthesia and analgesia (EA) is beneficial for postoperative cancer outcomes remains controversial and we conducted this historical cohort study to evaluate the association between EA and long-term outcomes following surgery for renal cell carcinoma (RCC). We collected patients receiving RCC surgery from 2011 to 2017 and followed up them until February 2020. Patient attributes, surgical factors and pathological features were gathered through electronic medical chart review. The association between EA and recurrence-free and overall survival after surgery was evaluated using Cox regression models with inverse probability of treatment weighting (IPTW) to balance the observed covariates. The median follow-up time for the 725 included patients was 50 months (interquartile range: 25.3–66.5) and 145 of them (20%) received perioperative EA. We demonstrated EA use was associated with better recurrence-free survival [IPTW adjusted hazard ratio (HR): 0.64, 95% confidence interval (CI): 0.49–0.83, *p* < 0.001] and overall survival [IPTW adjusted HR: 0.66, 95% CI: 0.49–0.89, *p* = 0.006] in patients receiving surgical resection for RCC. More prospective studies are needed to verify this connection between EA and superior cancer outcomes after RCC surgery.

## Introduction

Although life expectancy is increasing along with the progression and improvement of medical care, cancer remains one of the leading causes of death around the world and cancer treatment is still a great challenge in contemporary medicine (1). Surgical intervention is the mainstay treatment for the control and cure of most solid tumors but postoperative local or distant metastasis, which causes 90% of deaths, remains a common reason for morbidity and mortality in cancer patients (2, 3).

It should be noted that the perioperative period is associated with an increased formation of new metastatic foci and accelerated growth of micrometastatic disease (2). Surgical procedures themselves also suppress the host's immunity which is inhibiting pre-existing micro-metastases, and manipulation during surgery can disseminate cancer cells which are shed from the primary tumor to the blood stream or lymphatic system intraoperatively (4). Moreover, surgery can directly activate the hypothalamic-pituitary-adrenal axis and sympathetic nerve system to increase the levels of catecholamine, prostaglandins and acute inflammatory cytokines (interleukin-6, interleukin-8) that further suppress the cytotoxic activity of macrophages and natural killer (NK) cells (5).

Also, accumulating evidence shows that anesthetic intervention and analgesia could affect the pathophysiological processes associated with long-term cancer outcomes (6). Since immunity plays a major role in cancer progression (7), perioperative pain management could be very important for preventing surgery-induced immunosuppression. Perioperative epidural anesthesia and analgesia (EA) effectively attenuate neuroendocrine stress responses related to surgery, they also reduce intraoperative volatile anesthetics and opioid consumption by blocking noxious afferent inputs transmitted to the central nervous system, and further preserve host immunity (4, 7, 8). Previous studies have demonstrated that patients with perioperative EA had better prostate, ovarian, colon, gastro-esophageal and breast cancer outcomes compared with those without (7, 9). Nevertheless, few studies have investigated the association between EA and postoperative outcomes after surgery for renal cell carcinoma (RCC) (8).

To fill this gap, we hypothesized that EA is beneficial to long-term outcomes after curative surgery for RCC and we conducted this retrospective study to evaluate the association between EA and cancer recurrence or overall survival. We used a novel propensity weighted analysis to promote analytical power and to reduce potential confounding effects by incorporating important prognostic factors in the analysis. Sensitivity analysis using the two other regression approaches was also employed to ensure the consistency of the estimated results.

## Methods

### Setting and Patient Selection

**Figure 1:**
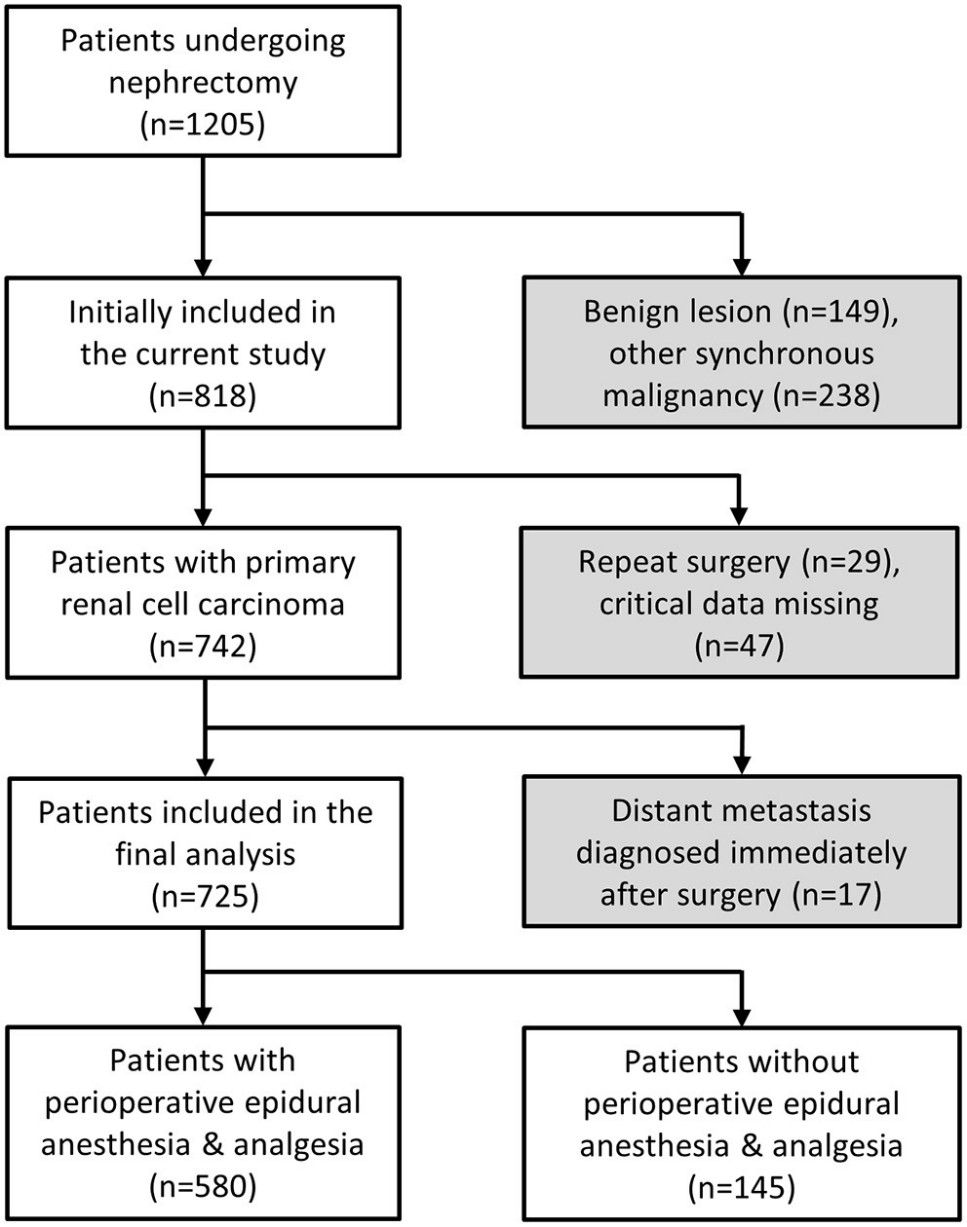
Flow diagram for patient selection.

The current study was approved by the Institutional Review Board (IRB) of the Taipei Veterans General Hospital, Taipei, Taiwan (IRB-TPEVGH no. 2018-06-009CC, Jul 2018), and written informed consent was waived by the IRB of the Taipei Veterans General Hospital. All methods were performed in accordance with the relevant guidelines and local regulations. Patients who underwent curative surgery for RCC between January 1st 2012 and December 31st 2017, as determined by reviewing the electronic medical records at our hospital, were included in the study. The exclusion criteria were: patients with benign pathological reports, non-RCC, reoperation for metastasis lesions, missing pathological data or perioperative pain management (Figure 1). All included patients were further classified into two groups based on whether they received perioperative EA or not. The reasons why patients did not receive EA included contraindications to EA, failed epidurals and the preference of the anesthesiologist, surgeon or patient, etc. For those who did not receive perioperative EA, intravenous patient-controlled analgesia with morphine was used to control postoperative pain.

### Anesthetic Management

During general anesthesia, the induction of anesthesia consisted in intravenous fentanyl 1–3 μg/kg, propofol 1–2.5 mg/kg and a neuromuscular blocking drug of either cisatracurium 0.15–0.2 mg/kg or rocuronium 0.6–1.2 mg/kg to facilitate tracheal intubation. Anesthesia was maintained with sevoflurane 2–3 vol% or desflurane 6–8 vol%. All epidural catheters were inserted at the lower thoracic or high lumbar region (T10–L2). Patients scheduled to receive EA had an epidural catheter implanted preoperatively which was tested using 2% lidocaine 2 ml to ensure it functioned properly. A loading dose of 1–1.5% lidocaine with or without fentanyl 50 μg was given before the surgical incision, and then bupivacaine 0.25% was continuous infused at a rate of 5–10 ml/h intraoperatively depending on the hemodynamic stability. EA was administered with bupivacaine 0.0625% for postoperative pain management and typically maintained for 48–72 h. Patients who did not receive EA had intravenous patient-controlled analgesia (PCA) *via* an ambulatory infusion pump (Gemstar Yellow, Hospira, IL, USA) to deliver morphine with an infusion rate of 0.5–1.0 mg/h and a bolus dose of 1 mg with a lockout time of 5–10 mins.

### Postoperative Cancer Control

RCC staging was defined according to the American Joint Committee on Cancer 2010 tumor-node-metastasis cancer classification system (10). After the primary tumor resection, additional surgeries for metastatic disease were performed depending on the lesion locations, including hepatectomy, colectomy, IVC thrombectomy, etc. Postoperative surveillance was performed regularly at an outpatient clinic and followed the National Comprehensive Cancer Network guidelines (11).

### Data Collection

We reviewed the patient's electronic medical records and collected their demographic characteristics, including age, sex, body mass index (BMI), American Society of Anesthesiologists (ASA) physical status and Charlson comorbidity index (12). We also collected potential risk factors which might affect cancer prognosis, including cancer staging, pathological features (histologic tumor necrosis, capsular invasion, hilar vein invasion, renal sinus invasion), perioperative blood transfusion, minimal invasive or traditional open surgery, partial or radical nephrectomy and smoking. Current disease status and date of death were also obtained from the medical records. Local recurrence or distant metastasis was determined using imaging studies (computed tomography, magnetic resonance imaging, bone scan) or a tissue biopsy. The primary outcome was recurrence-free survival (RFS) which was defined as the time interval between the date of surgery and the discovery date of cancer recurrence or new metastatic foci. The secondary outcomes were overall survival (OS) and cancer-specific survival (CSS). All patients were followed until they were lost to follow-up, death or the 29th February 2020, whichever came first. For those without cancer progression, survival times were defined as the corresponding censored observation. Competing risk events were regarded as censoring in the analysis of cancer-specific survival.

### Statistical Analysis

All the patients were classified into the two groups depending on whether they received EA or not. Continuous and categorical variables are presented as the mean with standard deviation and count with the percentage, respectively. Logarithmic transformation was conducted to reduce the skewness of non-normal continuous variables. Standardized differences were used to evaluate balance in the collected variables between the two groups. The Kaplan-Meier method was used to compare the RFS and OS between the two groups. Univariate Cox regression analysis was also performed to assess the effects of EA and other covariates on RFS, OS and CSS. An inverse probability of treatment weighting (IPTW) method based on propensity scores was used to balance the distributions of the collected variables in the EA and non-EA groups and 1% of subjects at the end of weighting distribution were truncated to reduce the impact of large weights on the analytical results (13). Note that the propensity scores were generated from the logistic regression analysis (Supplementary Table S1) and reflected the probability of receiving EA given the collected variables. IPTW methodology weighted study subjects by the inverse probability of receiving EA or not to create a pseudo-population where the EA assignment is independent of the collected variables like randomization for unbiased estimation of average EA effects. Accordingly, weighted Cox regression analysis was applied to evaluate the association between EA and RFS, OS or CSS based on IPTW. For sensitivity analysis, all of the patients were further divided into the five equal groups using the quintiles of the generated propensity scores and a stratified Cox regression analysis was conducted to obtain a pooled hazard across the five strata to estimate the association of EA with RFS, OS and CSS. In addition, multivariable Cox regression analysis with a stepwise model selection strategy was used to identify independent predictors of RFS, OS and CSS, and to evaluate the effects of EA on these long-term outcomes. The significance level of all hypothesis testing was set at 0.05 and all the statistical analyses were conducted using SAS software, version 9.4 (SAS Institute Inc., Cary, NC, USA).

## Results

### Patient Characteristics

A total of 725 patients were included in the study. They had a median follow-up interval of 50 with an interquartile range of 25.3 to 66.5 months and of these patients, 145 (20%) received EA. In the original sample, patients in the EA groups tended to be younger and have a higher chance of receiving open surgery (Table 1). Compared with the non-EA group, more cases in the EA group received surgery before 2015. However, after IPTW the imbalances in these covariate distributions between the two groups were removed (Table 1).

**Table 1:** Patient demographics.

	**Original data**			**After IPTW**		
	**Non-EA group**	**EA group**	**SDD**	**Non-EA group**	**EA group**	**SDD**
	**(*n* = 580)**	**(*n* = 145)**		**(*n* = 725)**	**(*n* = 622)**	
Age	59 ± 14	56 ± 14	22.06	58 ± 14	58 ± 12	1.92
BMI	25.65 ± 4.10	25.39 ± 3.84	6.60	25.61 ± 4.10	25.68 ± 3.81	1.86
ASA physical status > 3	162 (27.9%)	38 (26.2%)	3.88	201 (27.7%)	168 (27.0%)	1.52
Charlson comorbidity index	4.08 ± 1.70	3.79 ± 1.76	16.93	4.03 ± 1.69	4.13 ± 1.78	5.84
Anesthesia time^*^	8.55 ± 0.36	8.57 ± 0.38	3.69	8.56 ± 0.36	8.58 ± 0.39	4.14
Intraoperative blood loss^*^	7.65 ± 1.89	8.03 ± 1.93	19.82	7.73 ± 1.90	7.65 ± 2.05	3.75
Sex, male	396 (68.3%)	100 (69.0%)	1.49	497 (68.6%)	450 (72.2%)	8.04
Smoking	134 (23.1%)	38 (26.2%)	7.21	172 (23.7%)	151 (24.2%)	1.13
Surgical year			42.61			9.87
<2015	281 (48.4%)	100 (69.0%)		381 (52.6%)	358 (57.5%)	
Packed RBC transfusion	131 (22.6%)	41 (28.3%)	13.09	173 (23.8%)	148 (23.8%)	0.07
Laparoscopic or robotic surgery	321 (55.3%)	12 (8.3%)	117.12	333 (45.9%)	238 (38.2%)	15.73
Partial nephrectomy	351 (60.5%)	86 (59.3%)	2.46	437 (60.3%)	368 (59.1%)	2.34
Cancer subtype			12.87			7.46
Clear cell	441 (76.0%)	102 (70.3%)		543 (74.9%)	486 (78.1%)	
Others^**^	139 (24.0%)	43 (29.7%)		182 (25.1%)	136 (21.9%)	
Fuhrman grade > 2	215 (37.1%)	56 (38.6%)	3.20	271 (37.4%)	201 (32.3%)	10.70
Tumor necrosis	190 (32.8%)	54 (37.2%)	9.41	242 (33.4%)	179 (28.7%)	10.17
Capsular invasion	61 (10.5%)	10 (6.9%)	12.87	71 (9.8%)	40 (6.4%)	12.21
Hilar vein invasion	104 (17.9%)	27 (18.6%)	1.78	131 (18.1%)	138 (22.2%)	10.37
Renal sinus invasion	70 (12.1%)	22 (15.2%)	9.06	93 (12.8%)	92 (14.7%)	5.57
Cancer stage			7.18			2.76
I	369 (63.6%)	87 (60.0%)		454 (62.7%)	389 (62.5%)	
II	40 (6.9%)	14 (9.7%)		54 (7.5%)	39 (6.3%)	
III	141 (24.3%)	30 (20.7%)		171 (23.6%)	150 (24.2%)	
IV	30 (5.2%)	14 (9.7%)		45 (6.2%)	43 (7.0%)	

*Values were mean ± SD or counts (percent), or median (interquartile range). Standardized difference (SDD) is the difference in mean or proportion divided by the pooled standard error, expressed as percentage; imbalance is defined as absolute value greater than 20 (small effect size)*.

* *On base-2 logarithmic scale*.

** *Other morphological types of RCC include chromophobe, papillary, Xp11.2 translocation, etc*.

*BMI, body mass index; EA, epidural anesthesia and analgesia; IPTW, inverse probability treatment weighting; RBC, red blood cell; SDD, standardized difference*.

### Recurrence-Free Survival

**Figure 2:**
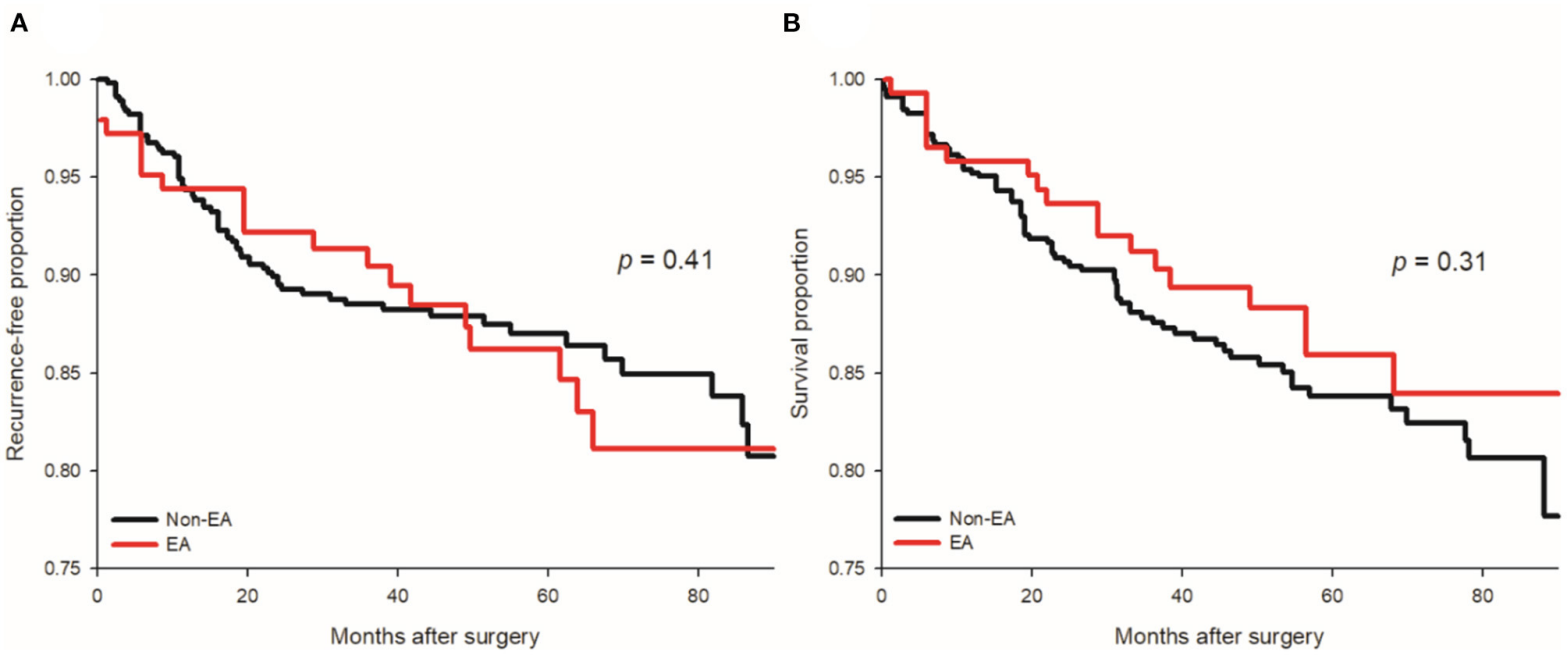
Kaplan-Meier curves for cancer recurrence and all-cause mortality of EA (epidural anesthesia and analgesia) and non-EA groups. No significant difference in cancer recurrence **(A)** or all-cause mortality **(B)** was found after renal cell carcinoma resection between the EA with non-EA groups.

The 5-year RFS rates were 81.6% [95% confidence interval (CI): 74.5–88.7%] and 78.7% (95% CI: 74.8–82.6%) in the EA and non-EA groups, respectively. No significant difference in RFS distribution was noted between the EA and non-EA groups (*p* = 0.408 by log rank test, Figure 2A). The crude hazard ratio (HR) of the EA group was 0.84 with a 95% CI of 0.55–1.28. However, after IPTW, the weighted Cox regression analysis demonstrated a significant association between the EA and a better RFS (adjusted HR = 0.64, 95% CI: 0.49–0.83; *p* < 0.001). For sensitivity analysis, multivariable regression analysis identified six independent prognostic factors for cancer recurrence, including perioperative transfusion, anesthesia time, tumor necrosis, capsular invasion, cancer staging and EA (adjusted HR = 0.62, 95% CI: 0.40–0.96, Table 2). Notice that the association between EA and superior RFS was significant in the quintile-stratified analysis (pooled HR = 0.64, 95% CI: 0.40–1.00; *p* = 0.05).

**Table 2:** Forward model selection for recurrence-free survival before weighting.

	**HR**	**95% CI**	***P***
Epidural analgesia	0.62	0.40–0.96	0.031
Anesthesia time^*^	2.12	1.29–3.47	0.003
Packed RBC transfusion	1.65	1.12–2.42	0.010
Tumor necrosis	2.40	1.63–3.54	<0.001
Capsular invasion	1.61	1.05–2.47	0.030
Cancer stage			<0.001
II vs. I	2.69	1.47–4.92	0.001
III vs. I	2.55	1.59–4.08	<0.001
IV vs. I	9.90	5.75–17.05	<0.001

* *On base-2 logarithmic scale. CI, confidence interval; HR, hazard ratio; RBC, red blood cell*.

### Overall and Cancer-Specific Survivals

The 5-year OS rates were 86.8% (95% CI: 80.5–93.1%) and 83.8% (95% CI: 80.3–87.3%) in the EA and non-EA groups, respectively. There was no significant association between EA and better OS in the univariate analysis (p = 0.305 by log rank test, Figure 2B). The crude HR of EA was 0.76 with a 95% CI of 0.45–1.29. EA was significantly associated with superior OS after IPTW (adjusted HR = 0.66, 95% CI: 0.49–0.89; *p* = 0.006) and in the quintile-stratified analysis (HR = 0.54, 95% CI: 0.31–0.94; *p* = 0.03). The multivariable analysis identified six independent prognostic factors of OS, including BMI, Charlson comorbidity index, anesthesia time, tumor necrosis, capsular invasion and cancer stage (Table 3). The association between EA and better OS after RCC surgery was not significant after the adjustment for these significant predictors (HR = 0.63, 95% CI: 0.37–1.07, *p* = 0.09). With respect to the CSS, significant associations between EA and better CSS were noted after IPTW (adjusted HR = 0.68, 95% CI: 0.49–0.94; *p* = 0.02) and in the quintile-stratified analysis (HR = 0.52, 95% CI: 0.28–0.97; *p* = 0.04). Six predictors of CSS were identified after the model selection processes and a significant protective effect of EA on CSS was also noted (HR = 0.49, 95% CI: 0.27–0.89, *p* = 0.02, Table 4).

**Table 3:** Forward model selection for overall survival before weighting.

	**HR**	**95% CI**	***p***
Epidural analgesia	0.63	0.37–1.07	0.086
BMI	0.93	0.88–0.99	0.025
Charlson comorbidity index	1.17	1.06–1.29	0.003
Anesthesia time^*^	3.15	1.84–5.39	<0.001
Tumor necrosis	2.83	1.75–4.60	<0.001
Capsular invasion	1.75	1.05–2.91	0.031
Cancer stage			<0.001
II vs. I	2.33	1.09–4.96	0.028
III vs. I	1.78	0.99–3.22	0.054
IV vs. I	11.44	6.26–20.94	0.000

* *On base-2 logarithmic scale. CI, confidence interval; HR, hazard ratio*.

**Table 4:** Forward model selection for cancer-specific survival before weighting.

	**HR**	**95% CI**	***P***
Epidural analgesia	0.45	0.27–0.89	0.011
Anesthesia time^*^	2.72	1.46–4.91	0.001
Packed RBC transfusion	1.86	1.12–3.05	0.016
Tumor necrosis	3.48	1.97–5.95	<0.001
Capsular invasion	1.91	1.15–3.32	0.017
Cancer stage			<0.001
II vs. I	2.41	0.87–5.27	0.060
III vs. I	2.56	1.17–4.37	0.007
IV vs. I	13.51	5.68–23.00	<0.001

* *On base-2 logarithmic scale. CI, confidence interval; HR, hazard ratio; RBC, red blood cell*.

## Discussion

In recent decades, perioperative management has been identified as a factor that could impact cancer outcomes by altering the microenvironment and it has been receiving more clinical attention (14). All tissue trauma, including the sterile dissection carried out by surgeons, and inflammation have been associated with tumor progression (2). This study demonstrated the hypothetical benefits of perioperative EA for long-term cancer control and survival in patients following RCC resection. To the best of our knowledge, the current study is the largest comparative epidural study to date which has investigated the association between EA and long-term outcomes after RCC surgery. The current study had several strengths, including a relatively large sample size, and the fact that we took more prognostic and pathologic factors into account. We also used sound analytical approaches such as IPTW and other regression-based sensitivity analyses to ensure the consistency of the estimated results (9). Charlson comorbidity index was also used to control for the potential influence of comorbidity severity on the outcomes of interest in the analysis (15). All these efforts were used to try and provide more precise and reliable estimated results to determine the actual association between EA and RFS or OS after curative surgery for RCC.

Although opioids are widely used to control postoperative pain, they are believed to have negative effects on the immune system (8). The evidence from clinical observational studies indicates that opioids suppress cellular and humoral immunity, promote angiogenesis, and enhance progression of metastatic disease (2). Overexpression of μ-opioid receptors on cancer cells is observed and associated with angiogenesis and oncogenic signaling (4). Perioperative EA is administered near the nerve roots to block sensory and sympathetic nerves. It attenuates the neuroendocrine stress responses of the hypothalamic-pituitary-adrenal axis and sympathetic nervous system activation (4) and minimizes volatile agent and opioid consumption (7, 9). Therefore, it has been suggested that EA preserves immune function and prevents cancer recurrence after curative surgery but previous studies have reported inconsistent results (7, 14). Zimmitti et al. reported improved RFS but not OS in patients receiving general anesthesia with EA compared to those without after hepatic resection for colorectal liver metastases (16). However, the study did not take pre-existing medical conditions, perioperative blood transfusion or pathological features into account. Myles et al. (17) found no significant difference in RFS or OS between the EA and non-EA groups following abdominal cancer surgeries in a *post hoc* review of randomized control trials. A systemic review and meta-analysis revealed a lower risk of OS but not cancer recurrence among patients with perioperative regional anesthesia and analgesia (9). A recent study compared perioperative systemic analgesia (SA group) with perioperative EA in addition to systemic analgesia (EA group) following surgical resection of localized RCC, which suggested that EA usage was associated with a significantly improved OS but did not significantly impact cancer-specific survival (8). Accordingly, more prospective studies are needed to elucidate the associations between EA and long-term outcomes after curative cancer surgeries.

During cancer development, circulating tumor cells may leave the primary tumor and form clinically undetectable metastatic foci (2). Micrometastases remain in an immunologic equilibrium between tumor cell proliferation and host immunity (4). However, a cascade of local, systemic cellular and humoral inflammation events may reduce the ability of the host immune system to detect and eradicate cancer cells and could help to disseminated cancer cells which survive the host's defensive mechanisms (2, 14). Clinical evidence shows that tissue trauma caused during surgery can accelerate subsequent neoplastic disease (2, 6). Moreover, an experimental trial reported that the more extensive the surgery is the more potential there is for postoperative inflammation and complications, which further increase the recurrence rate (18). Some studies have suggested that open cancer surgery was associated with shorter disease-free survival compared with minimally invasive surgery, which limited surgical trauma (2, 19), however our investigation did not support the beneficial effects of minimally invasive surgery and partial nephrectomy compared with open surgery and radical nephrectomy, respectively. Notice that similar findings were also noted in another two studies which investigated oncological outcomes in patients undergoing minimally invasive surgery compared with open surgery for clinical T2 RCC and locally advanced RCC, respectively (20, 21). Similar findings were also noted in another study comparing partial or radical nephrectomy for clear cell RCC larger than 7 cm (22).

Interestingly, we also noted that a longer anesthesia time was associated with worse RFS and OS in the stepwise regression analysis. Singh et al. (23) had similar findings in an analysis of minimally-invasive surgeries for endometrial cancer. In their study, longer operative time was also associated with increased medical, surgical and overall complication rates. In fact, longer anesthesia time, as a surrogate for longer surgical time, may reflect the underlying aggressiveness of the disease or the complexity and difficulty of the surgery, or both. Since we have taken miscellaneous surgical and oncological factors into consideration to reduce confounding effects, anesthesia time is highly suspected as an independent risk factor of cancer recurrence and mortality in patients receiving RCC surgery.

Still, there are other factors which may have an effect on long-term cancer outcomes, including the use of steroids (14), non-steroidal anti-inflammatory drugs (4, 18, 19) and systemic lidocaine (4, 14), hypothermia (2, 14, 18), postoperative infections (2), blood transfusions (2, 14), etc. Red blood cells (RBC) are commonly given to cancer patients before, during or after major surgery for a number of different reasons. Although the value of blood transfusions for saving lives is indisputable (24), blood-component therapy can induce negative effects on the recipients' immune system (25), a condition called “transfusion-related immunomodulation” (25, 26). The detrimental effects of immunomodulation are thought to have an association with systemic inflammation (26, 27) and various immunologic changes, including inhibition of cytotoxic cell activity, and immunosuppressive prostaglandin release (26). In the sensitivity analysis, we observed that perioperative packed RBC transfusion was associated with a worse RFS in the multivariable regression analysis. Abu-Ghanem et al. (28) also reported that transfusion reduced RFS, CSS and OS in patients undergoing nephrectomy for RCC. Tsivian et al. (29) found that perioperative blood transfusion was independently associated with worse oncological outcomes for localized RCC after curative surgery and that the recipients were associated with roughly a two-fold increase in metastatic progression, all-cause and RCC-specific mortality. Moreover, negative clinical outcomes were also observed in colon (26, 27) and esophageal (30) cancer patients who received transfusion during curative-intent surgeries. Based on these findings, it has been suggested that blood transfusion can influence the different stages of tumor development including initiation, promotion, malignant conversion, invasion and metastasis (24). To reduce the potential confounding of perioperative blood transfusion on the outcomes of interest, the IPTW methodology was used to balance the exposure of transfusion in both the EA and non-EA groups.

The current study had several limitations. First, patients were not randomized to either group, they instead received EA depending on the preference of the patient, the surgeon or the anesthesiologist. Second, the influence of unmeasured covariates such as dose of opioid, local anesthetics or volatile agents and non-steroidal anti-inflammatory drugs use on cancer outcomes cannot be evaluated due to a lack of available data. Third, the clinical outcomes of loss to follow-up patients are unknown and the last observed censoring time was used in the analysis, which may have affected the results.

In conclusion, we demonstrated an association between perioperative EA use and better RFS and OS in patients undergoing curative surgery for RCC. Future prospective studies and randomized clinical trials with careful design are needed to confirm this relationship between EA and cancer outcomes after curative surgery for RCC and to elucidate the underlying mechanisms.

## Data Availability Statement

The data analyzed in this study is subject to the following licenses/restrictions: the dataset is only available after the approval of the IRB of Taipei Veterans General Hospital. Requests to access these datasets should be directed to irbopinion@vghtpe.gov.tw.

## Ethics Statement

The studies involving human participants were reviewed and approved by Institutional Review Board (IRB) of the Taipei Veterans General Hospital, Taipei, Taiwan (IRB-TPEVGH No. 2018-06-009CC, Jul 2018). Written informed consent for participation was not required for this study in accordance with the national legislation and the institutional requirements.

## Author Contributions

F-YY: data collection and manuscript preparation. S-PL: data verification and interpretation. T-PL: data verification and manuscript revision. W-KC: statistical review and manuscript revision. K-YC: study design, data analysis, manuscript preparation, and critical content editing. All authors contributed to the article and approved the submitted version.

## Funding

This study was supported by grants from Taipei Veterans General Hospital, Taipei, Taiwan (V109C-063), the Ministry of Science and Technology, Taipei, Taiwan (MOST108-2511-H-075-001), and the Anesthesiology Research and Development Foundation, Taipei, Taiwan (ARDF10804).

## Conflict of Interest

The authors declare that the research was conducted in the absence of any commercial or financial relationships that could be construed as a potential conflict of interest.

## Publisher's Note

All claims expressed in this article are solely those of the authors and do not necessarily represent those of their affiliated organizations, or those of the publisher, the editors and the reviewers. Any product that may be evaluated in this article, or claim that may be made by its manufacturer, is not guaranteed or endorsed by the publisher.
