# Supplementary material for: Association Between Epidural Analgesia and Cancer Recurrence or Survival After Surgery for Renal Cell Carcinoma: A Propensity Weighted Analysis
Source: Front Med (Lausanne). 2022 Jan 14;8:782336. doi: 10.3389/fmed.2021.782336 (PMC8795372; doi:10.3389/fmed.2021.782336)
Supplement: Supplementary file 1 [file Table_1.PDF]

**S1 Table. The results of logistic regression analysis for inverse probability treatment weighting**

| | OR | 95% CI | *p* |
|---|---|---|---|
| Sex | 0.90 | 0.57 ~ 1.43 | 0.666 |
| BMI | 1.00 | 0.94 ~ 1.05 | 0.878 |
| Charlson comorbidity index | 0.94 | 0.82 ~ 1.06 | 0.303 |
| Smoking | 1.37 | 0.83 ~ 2.25 | 0.216 |
| Surgical year ≥ 2015 vs. < 2015 | 0.44 | 0.28 ~ 0.68 | < 0.001 |
| Anesthesia time* | 1.15 | 0.58 ~ 2.28 | 0.686 |
| Intraoperative blood loss* | 0.94 | 0.81 ~ 1.11 | 0.476 |
| Packed RBC transfusion | 0.97 | 0.55 ~ 1.70 | 0.905 |
| Laparoscopic or robotic surgery | 0.07 | 0.04 ~ 0.13 | < 0.001 |
| Partial nephrectomy | 0.98 | 0.58 ~ 1.66 | 0.935 |
| Clear cell | 0.76 | 0.47 ~ 1.22 | 0.259 |
| Fuhrman grade > 2 | 1.02 | 0.64 ~ 1.62 | 0.945 |
| Tumor necrosis | 1.05 | 0.65 ~ 1.71 | 0.834 |
| Capsular invasion | 0.47 | 0.20 ~ 1.09 | 0.078 |
| Hilar vein invasion | 1.19 | 0.55 ~ 2.59 | 0.657 |
| Renal sinus invasion | 0.92 | 0.44 ~ 1.91 | 0.818 |
| Cancer stage | | | 0.893 |
| II vs. I | 0.99 | 0.45 ~ 2.16 | 0.979 |
| III vs. I | 1.00 | 0.43 ~ 2.35 | 0.992 |
| IV vs. I | 1.40 | 0.48 ~ 4.05 | 0.535 |

*On base-2 logarithmic scale

BMI = body mass index; CI = confidence interval; OR = odds ratio; RBC = red blood cell.
